# Development of a Broad-Spectrum Antigen-Capture ELISA Using Combined Anti-p26 Polyclonal and Monoclonal Antibodies for Detection of Equine Infectious Anemia Virus

**DOI:** 10.3390/microorganisms13071500

**Published:** 2025-06-27

**Authors:** Haibing Liang, Bingqian Zhou, Zhe Hu, Xiaoyu Chu, Xuefeng Wang, Cheng Du, Xiaojun Wang

**Affiliations:** 1 State Key Laboratory for Animal Disease Control and Prevention, Harbin Veterinary Research Institute, Chinese Academy of Agricultural Sciences, Harbin 150069, China; lianghaibing0209@163.com (H.L.); zhoubingqian9901@163.com (B.Z.); huzher@126.com (Z.H.); chuxiaoyu613@163.com (X.C.); wangxuefeng@caas.cn (X.W.); 2 Institute of Western Agriculture, The Chinese Academy of Agricultural Sciences, Changji 831100, China

**Keywords:** EIAV, AC-ELISA, p26

## Abstract

Equine Infectious Anemia Virus (EIAV) poses significant diagnostic challenges due to its genetic variability and the limitations of conventional nucleic acid detection methods. This study developed an antigen-capture, enzyme-linked immunosorbent assay (AC-ELISA) for the detection and quantification of the EIAV capsid protein p26. The assay utilized a monoclonal antibody (1G11) specific to the p26 protein as the capture antibody and a polyclonal antibody as the detection antibody, forming a highly specific and sensitive detection system. Under optimized conditions, the detection limit of the AC-ELISA was 1.95 ng/mL, with a good linear relationship observed between 1.95 ng/mL and 60.5 ng/mL of p26 protein. Additionally, the AC-ELISA effectively distinguished EIAV from other equine viruses, including equine herpesvirus 1 (EHV-1), equine arteritis virus (EAV), and equine influenza virus (EIV), without cross-reactivity. Importantly, the AC-ELISA demonstrated the ability to detect multiple EIAV strains, including virulent strains, attenuated strains, and strains from other countries, highlighting its broad applicability across diverse EIAV isolates. Compared to western blot and reverse transcriptase assays, the AC-ELISA exhibited higher sensitivity and strong correlation in quantifying the EIAV p26 protein. The assay is simple, rapid, and cost-effective, making it suitable for both laboratory research and clinical applications. It provides a powerful tool for EIAV detection and quantification, supporting future vaccine development and clinical trials.

## 1. Introduction

Equine infectious anemia (EIA), caused by the equine infectious anemia virus (EIAV), is an infectious disease that infects members of the *Equidae* family, including horses, donkeys, mules, and zebras [1,2]. It is classified as a notifiable disease by the World Organization for Animal Health (WOAH) and is characterized by recurrent fever, thrombocytopenia, anemia, rapid weight loss, and edema in the lower limbs and abdomen [3,4]. EIAV is a member of the lentivirus family and belongs to the family *Retroviridae*. It has been extensively studied for its unique integration characteristics within the host genome, providing valuable insights into lentivirus research, including human immunodeficiency virus (HIV) [5,6,7]. To date, EIA has remained a global disease with epidemic occurrences and occasional outbreaks in various regions [2,3,8,9]. The transmission of EIAV among horses occurs naturally through the bites of blood-feeding insects, and iatrogenic transmission occurs via the reuse of injection needles, syringes, and surgical equipment, as well as through the direct administration of infected blood and plasma products [8]. Consequently, EIA now poses a substantial economic threat to the global equine industry, driven by diverse transmission routes, a growing population of horses and donkeys, and their increased mobility. Therefore, the prevention and control of EIA should not be taken lightly.

Highly sensitive diagnostic methods with broad-spectrum recognition capabilities for diverse prevalent strains are indispensable for the prevention and control of animal diseases. However, the lack of specific disease characteristics impairs EIA diagnosis based on clinical signs or clinicopathologic findings [9], and the diagnosis of EIA therefore primarily relies on laboratory testing. To date, several laboratory testing methods have been established for EIAV, including serological tests (agar gel immunodiffusion [AGID] and enzyme-linked immunosorbent assay [ELISA]), nucleic acid detection by PCR, and antigen detection of viral antigen by AC-ELISA. Furthermore, virus isolation is not used as a routine diagnostic test for EIA [10,11]. Although AGID has been officially recognized as the “gold standard” for serological testing by the WOAH and many countries worldwide, this method exhibits relatively low sensitivity, requires a prolonged detection period exceeding 24 h, and relies on subjective readings, which precludes automation [10]. This has led to the method being regarded as suitable primarily for detecting the history of exposure of equine populations to EIAV. Besides AGID, another serological detection method for EIAV is the ELISA assay, including iELISA and cELISA, based on the p26 antigen of EIAV, which is commonly used for EIAV screening [11]. However, seroconversion in infected equids is not easily detectable as a characteristic of lentiviral infection, and antibody levels remain very low even after 45 or up to 157 days of exposure to EIAV [12]. The limitations above significantly impact on the effectiveness and practicality of serological detection methods for the diagnosis of EIAV.

**Figure 1:**
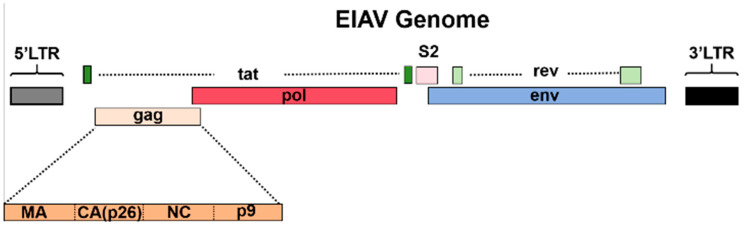
Genome structure of EIAV. The genome of EIAV consists of three genes encoding structural proteins (gag, pol, env) and three genes encoding accessory proteins (rev, tat, S2), with a total length of approximately 8.2 kb. The precursor protein of the structural protein gag, Pr55gag (p55), has a molecular mass of about 55 kDa. As the viral particle matures, Pr55gag is cleaved by the protease encoded by the pol gene, producing four major structural proteins: capsid protein (CA, p26), matrix protein (MA, p15), nucleocapsid protein (NC, p11), and core protein (p9).

**Figure 2:**
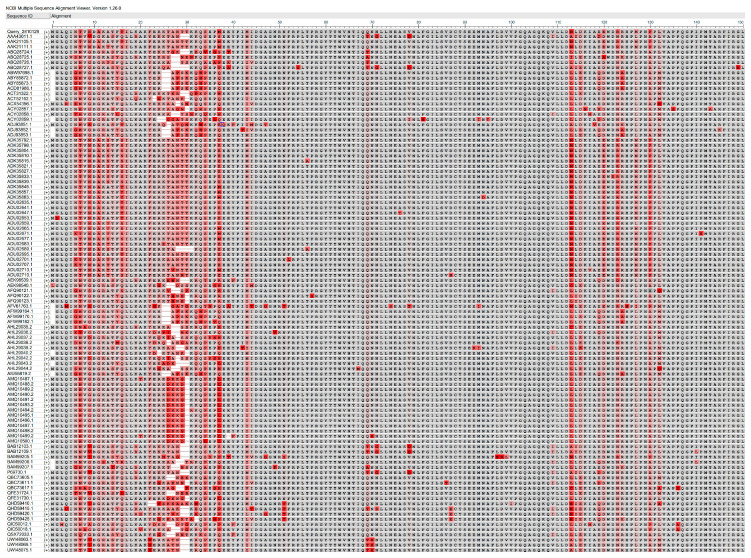

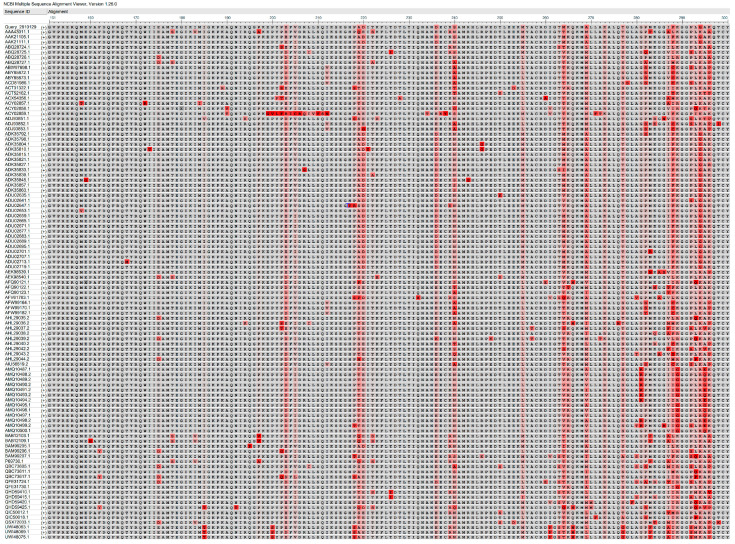
Comparison of amino acid sequences of the p26 protein among different EIAV strains. Darker red colors indicate lower consistency.

Pathogen detection methods (PCR, which is recommended by the WOAH, and AC-ELISA) also target p26 [13,14,15,16]. EIAV is one of the simplest lentiviruses, with a genome that is approximately 8.1 kb, and encodes three main structural proteins (gag, pol, and env) and three non-structural proteins (tat, rev, and S2) (Figure 1) [17,18,19]. The main antigen for EIAV detection is the capsid protein p26, a product of the cleavage of the gag precursor polyprotein (p55) by the viral protease during the maturation of the viral particle. Protein p26 is the most abundant protein in viral particles and serves as the primary target of the humoral immune response [20,21]. Although the gag and pol genes of EIAV are relatively more conserved than the LTR and Env sequences [2,22,23], p26 still exhibits considerable genomic variability among different EIAV isolates (Figure 2) [24,25,26]. Polymorphism of p26 not only affects the efficiency of serological diagnosis but also limits the effectiveness of antigen-based methods designed to target this region due to mismatches between the primers or antibodies and their target. Detection is [1,8,27]. PCR methods also suffer from other drawbacks such as procedural complexity, high time consumption, and a high rate of false-positives, making them inadequate for the diagnosis of EIAV in many situations [28].

The AC-ELISA targeting p26 with monoclonal antibody (mAb) and polyclonal antibody (pAbs) was prepared in this study. This method is simple to operate and cost-effective, requiring no complex equipment, and is suitable for rapid screening, providing an efficient tool for the basic research of EIAV. In the future, following optimization of the antibody library, we expect to achieve broader strain coverage, providing key technical support for EIAV evolution research and vaccine design.

## 2. Materials and Methods

### 2.1. Animals

The New Zealand white rabbits aged 6–8 weeks used in this study were obtained from the Changsheng (Liaoning) company (Shenyang, China). The physical procedures were performed under anesthesia to minimize pain and distress, in accordance with the recommendations of the Ethics Committees of HVRI. The Animal Ethics Committee approval number is Heilongjiang-SYXK (Hei) 240122-04-GR.

### 2.2. Plasmids and Viruses

EIAV virulent strains from China (EIAV_DLV34_) and the USA (EIAV_UK_, GenBank: AF016316.1,a molecular clone originating from the EIAV_wyo_) [24] were stored in our lab at −80 °C [29]. The attenuated EIAV infectious clone pCMV3-8 rescued virus (vCMV3-8) was stored in our lab at −80 °C [30]. Equine herpesvirus 1 (EHV-1) was isolated from infected tissue samples through cell culture propagation and stored in our lab at −80 °C. Equine influenza virus (EIV) was isolated from infected tissue samples through cell culture propagation (chicken embryo propagation) and stored in our lab at −80 °C. Equine arteritis virus (EAV) strain (Bucyrus strain) was kindly provided by Professor Zhang Nianzu from the Yunnan Tropical and Subtropical Animal Viral Disease Laboratory and stored at −80 °C [31]. All of these strains were titered by TCID_50_.

An EIAV pseudo-virus that expresses luciferase (EIAV-luc) was constructed by modifying a three-plasmid EIAV transfection system. HEK293T cells were co-transfected with pONY8.1-luc, pEIAV-GagPol, and pcDNA-env. The pseudo-virus was collected at 48 h post transcription, centrifuged at 1000 rpm for 10 min to remove cell debris, filtered through a 0.45-mm-pore filter unit (Thermo Fisher, Shanghai, China), and stored at −80 °C. These pseudo-virus particles cannot proceed to the next round of replication, and real-time qPCR analysis revealed an initial pseudo-virus particle concentration of 482,671 genomic copies per microliter (copies/μL) [32]. EIAV-luc served as a viral standard material in this study because of its biosafety and controllability.

### 2.3. Monoclonal Antibody and Polyclonal Antibody

A monoclonal antibody, 1G11, was prepared in our lab by immunizing mice with purified p26 protein [31]. The linear B-cell epitope of the 1G11 mAb is located in the amino acid sequence ^199^KNAMRHLRPEDTLEEKMYAC^218^. The 1G11 epitope can be recognized by anti-EIAV sera from different regions, including China, the USA, and Argentina. Meanwhile, 1G11 mAb is able to react with the mutants of almost all the EIAV strains [33].

The p26-His protein was purified according to a previously published protocol [31] and was then administered via intramuscular injection (im) to 6-week-old female New Zealand white rabbits to generate custom p26 polyclonal antibodies, following a proprietary procedure [34]. For the first immunization, rabbits were injected with purified p26 protein (1.0 mg/each) emulsified in complete Freund’s adjuvant (Sigma, St. Louis, MO, USA). Subsequent booster doses of the same amount, also emulsified in incomplete Freund’s adjuvant (Sigma, St. Louis, MO, USA), were administered every 2 weeks. The levels of p26-specific antibodies in the serum of the immunized rabbits were evaluated using indirect ELISA [31]. When the titers of p26-specific antibodies reached 1:1,000,000, the rabbits were euthanized using methods that comply with animal ethics, and all sera were aliquoted and frozen at −80 °C.

### 2.4. Antigen Captured ELISA (AC-ELISA)

To optimize the amounts of coating antigen used, a checkerboard assay was conducted as follows: The coating protein 1G11 mAb was diluted to six concentrations (12.5 ng/well, 25 ng/well, 50 ng/well, 100 ng/well, 200 ng/well, and 400 ng/well). The pAb (detection antibody) was diluted to three concentrations (1:3200, 1:6400, and 1:12,800). Then, 5% skimmed milk (Biosharp, Hefei, China), 5% newborn calf serum sterile (NBS) (Ausbian, Melbourne, VIC, Australia), 5% bovine serum albumin (BSA) (Biosharp, Hefei, China), and 1% BSA were used to determine the optimum block buffer. The optimum reaction conditions for use in this AC-ELISA were selected based on the minimum ratio of OD_450_ nm values between the positive and negative controls (P/N). The standard AC-ELISA procedure was as follows: A 96-well plate was coated with 1G11 mAb (400 ng/well) in PBS buffer (0.1 M, PH 7.4) at optimum concentrations at 2–8 °C for 12–16 h. After washing three times with 200 μL PBST (PBS with 0.05% Tween), the plate was blocked with the optimum block buffer in an incubator at 37 °C for two hours and then again washed three times with PBST. Next, the samples were added to the wells (100 μL/well), and the plate was put into an incubator at 37 °C for 3 h. Subsequently, the plates were incubated at room temperature for 1 h with 100 µL/well rabbit polyclone antibody. After washing again, goat anti-rabbit immunoglobulin G-peroxidase conjugate (Sigma, St. Louis, MO, USA) (dilution ratio 1:2000) was added to each well and incubated for 40 min at 37 °C. After washing again, the plate was stained with TMB at 15–25 °C for 10 min and the reaction was stopped with the addition of 50 μL 2 M H_2_SO_4_. Finally, the OD_450_ nm value of the plate was measured using enzyme calibration (Biotek, Winooski, VT, USA). A standard curve was generated using the known concentrations of the p26 standards, and the corresponding concentrations of the samples were calculated based on this standard curve. The cutoff value of AC-ELISA was determined by calculating the mean absorbance value of 10 EIAV negative serum samples plus 3 × standard deviations (S.D). We calculated a cutoff value of 0.15.

### 2.5. Determination of the Analytical Sensitivity and Specificity of the AC-ELISA

The analytical sensitivity of the AC-ELISA was determined using a serial dilution of p26 protein and EIAV-luc, which was diluted to ratios of 1:8, 1:16, 1:32, 1:64, 1:128, 1:256, 1:512, 1:1024, 1:2048, 1:4096, and so on with PBS. To evaluate the analytical specificity of the AC-ELISA, EIAV-luc was used as a positive control, and EHV-1, EAV, EIV, and dilution buffer were used as negative controls, and these were simultaneously tested. To assess the broad-spectrum recognition of different EIAV strains using the AC-ELISA, EIAV_UK_, EIAV_DLV34_, and vCMV3-8 were tested simultaneously with positive and negative controls.

### 2.6. Reverse Transcriptase Assay

A reverse transcriptase assay using a Reverse Transcriptase Assay Kit (ROCHE, Basel, Switzerland) was conducted following the manufacturer’s instructions [35]. Briefly, the assay involves lysing retroviruses in 40 μL of buffer at 15–25 °C for 30 min, performing reverse transcription with 20 μL of reaction mix at 37 °C for 1–15 h, and conducting ELISA by adding 60 μL of samples to MP modules, incubating with anti-digoxigenin-peroxidase and 2,2′-azino-bis(3-ethylbenzothiazoline-6-sulfonic acid (ABTS) substrate, washing 5 times, and measuring absorbance at 405 nm.

### 2.7. Real-Time PCR for EIAV-Luc

We used a SYBR green fluorescent dye (TakaRa, Dalian, Liaoning, China)-based real-time PCR method to determine the copies of the EIAV-luc. Since the EIAV-luc genome retains only essential elements like the *rev* gene (while lacking most wild-type genes) to boost production efficiency, we identified optimal primers within the *rev* gene region through primer screening. The primer sequences were as follows: *rev*-F: 5′-GGCACTCAGATTCTGCGG-3′; *rev*-R: 5′-CTGTAGGATCTCGAAC-3′. The size of the amplification product is 116 bp. Additionally, the *rev* plasmid (pcDNA3.1-*rev*) was used to prepare the standard curve and assess the copy of the EIAV-luc genome. The reaction conditions were 95 °C for 10 min, followed by 40 cycles of 95 °C for 5 s and 60 °C for 15 s.

### 2.8. Western Blotting

To characterize the p26 protein recognized by mAbs 1G11 and pAb, the p26-His protein, vCMV3-8, and EIAV-luc samples were initially separated on SDS-PAGE gels with a concentration gradient of 12%. Subsequently, they were transferred onto an NC membrane and probed with mAbs 1G11 or pAb. The detection of the p26 protein was achieved using a DyLightTM 800-conjugated secondary antibody or DyLightTM 680-conjugated secondary (KPL, Gaithersbg, MD, USA), and the signals were visualized on a Licor Odyssey Clx imaging system.

## 3. Results

### 3.1. Preparation of the p26 Protein and Validation of Antibody Effectiveness

**Figure 3:**
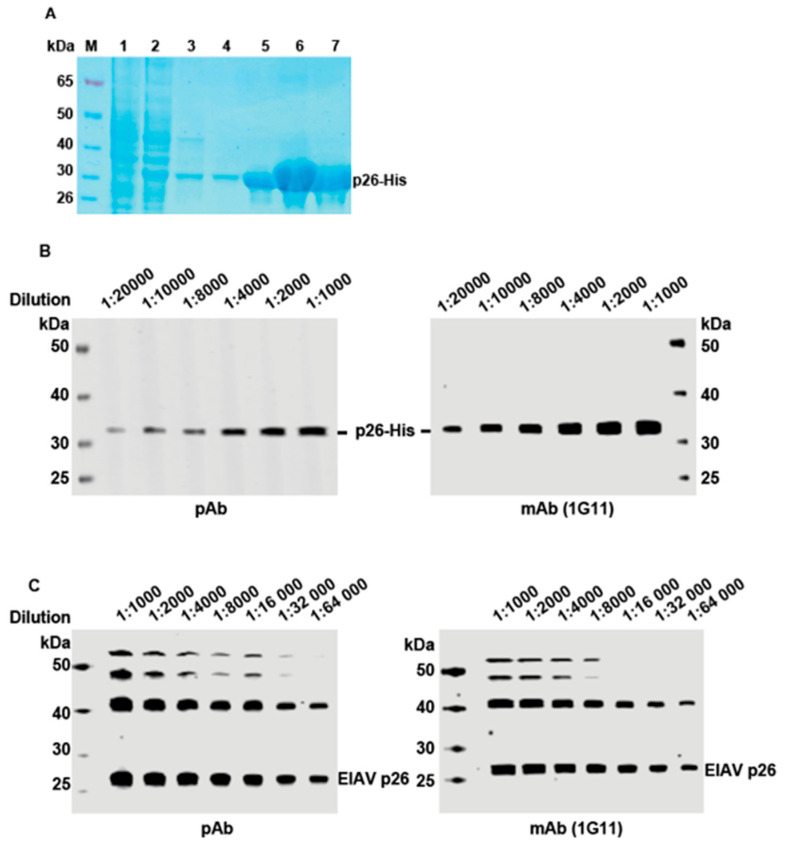
Preparation of antibodies and validation of their specificity. (**A**) Purified p26 protein of EIAV with His tag M: protein maker; 1: uninduced; 2: induced with IPTG; 3–4: conducted with wash buffer; 5–7: purified His-p26 protein. (**B**) The samples of purified p26-His protein were diluted to various concentrations for the WB and detected by pAb and 1G11 mAb. (**C**) The vCMV3-8 was diluted to various concentrations for the WB and detected by pAb and 1G11 mAb.

The recombinant p26-His protein was purified using an Ni-NTA column (Figure 3A) with high purity and concentration. The specificity of the pAb generated in the immune sera and the 1G11 mAb was demonstrated with WB using both the p26 protein and vCMV3-8 (Figure 3B,C). The results showed that both pAb and 1G11 mAb were able to recognize the p26 antigen with high sensitivity.

### 3.2. Establishment of the AC-ELISA

**Figure 4:**
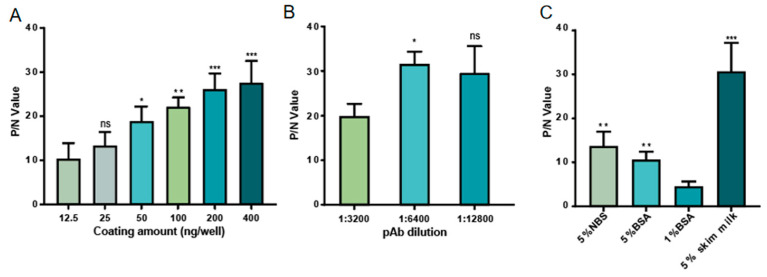
Establishment of the AC-ELISA. (**A**) Optimization of the coating concentration of the capture antibody. (**B**) Optimization of the dilution of pAb. (**C**) Optimization of the blocking buffer. Error bars indicate the SD from three independent experiments. *, *p* < 0.05; **, *p* < 0.01; ***, *p* < 0.001.

To develop a high-sensitivity AC-ELISA, it is necessary to optimize assay conditions, including coating concentration of the capture antibody, blocking buffer, and concentration of pAb. The results showed that the optimal 1G11 mAb coating concentration was 400 ng/well (Figure 4A), the optimal dilution of the pAb was 1:6400 (Figure 4B), and the most suitable blocking buffer was 5% skim milk (Figure 4C).

### 3.3. Specificity of the AC-ELISA

**Figure 5:**
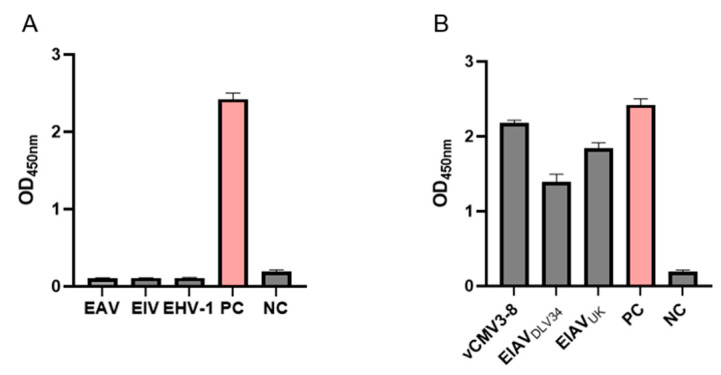
Specificity of the ELISA for p26. (**A**) Major viruses that infect equine were selected (EAV, EIV, EHV, and EIAV) to assess the specificity of AC-ELISA across different viruses. EIAV-luc as positive control, PC and dilution buffer as negative control, NC. (**B**) Different EIAV strains were selected to evaluate the broad-spectrum of p26 AC-ELISA.

To confirm the specificity of the p26 AC-ELISA assay, EHV-1, EAV, EIV, EIAV-luc (positive control, PC), and dilution buffer (negative control, NC) were analyzed using AC-ELISA as samples in separate reactions. As demonstrated in Figure 5A, EIAV-luc exhibited specific reactivity with no detectable cross-reactions against EHV-1, EAV, EIV, or NC. These results demonstrate that the assay is specific for detection of EIAV. To confirm that the novel AC-ELISA demonstrated broad-spectrum ability to detect different EIAV strains, the EIAV_DLV34_, EIAV_UK_, and vCMV3-8 strains were analyzed using AC-ELISA as samples in separate reactions [34]. As shown in Figure 5B, all strains exhibited a strong positive reaction, except for the negative control. This indicates that the AC-ELISA established in this study exhibits excellent recognition capability for different EIAV strains, while showing no cross-reactivity with other common equine viruses.

### 3.4. Analysis of the Sensitivity of the Novel AC-ELISA

**Figure 6:**
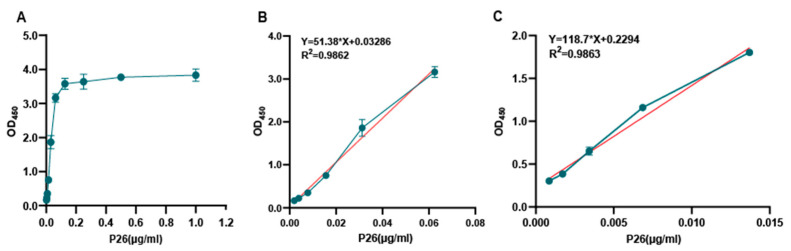
Sensitivity of the ELISA for p26. (**A**) Different concentrations of purified p26 protein with a serial two-fold dilution from 1 μg/mL to 0.002 μg/mL were tested. (**B**) The linear range of determination for EIAV p26 detection was 0.002–0.06 μg/mL. (**C**) Serially two-fold-diluted EIAV-luc was quantified by AC-ELISA. The amount of EIAV-luc in the minimum virus dilution (8×) with the OD value in the dynamic range was calculated using the linear equation of p26. There was also a good linear relationship from 1:8 to 1:4096 dilutions of EIAV-luc. The error bar shows the means of triplicate. Blue data points: Raw experimental measurements used to generate the calibration curve. Red regression line: Quantification of the relationship between Y-axis and X-axis.

Under optimized conditions, the sensitivity of the AC-ELISA for detecting EIAV p26 antigen was determined with two-fold serial dilutions of purified p26 protein with an initial concentration of 1 μg/mL. The minimum detection of p26 protein was 0.002 μg/mL (Figure 6A), and the linearity range of the AC-ELISA was obtained at the concentration of 0.002–0.06 μg/mL (Figure 6B). The resulting linear equation is y = 51.38x + 0.03286, and the correlation coefficient (R^2^) of the linear regression is higher than 0.98 (Figure 6B). For the quantification of EIAV-luc, the minimum virus dilution (8×) having OD_450_ values (3.554) within the dynamic range was found to agree with the corresponding concentration of diluted EIAV-luc [(3.554 − 0.0328)/51.38 = 0.069 μg/mL]. The detection limit of EIAV-luc was 4096 times the dilution of concentrated EIAV-luc. Furthermore, the linear formula of EIAV-luc was similar to that of p26 (Figure 6B). These results indicated that the sensitivity of the AC-ELISA for the detection of EIAV is consistent with that for the detection of the p26 protein.

### 3.5. Comparison of the Novel AC-ELISA and Other Methods

**Figure 7:**
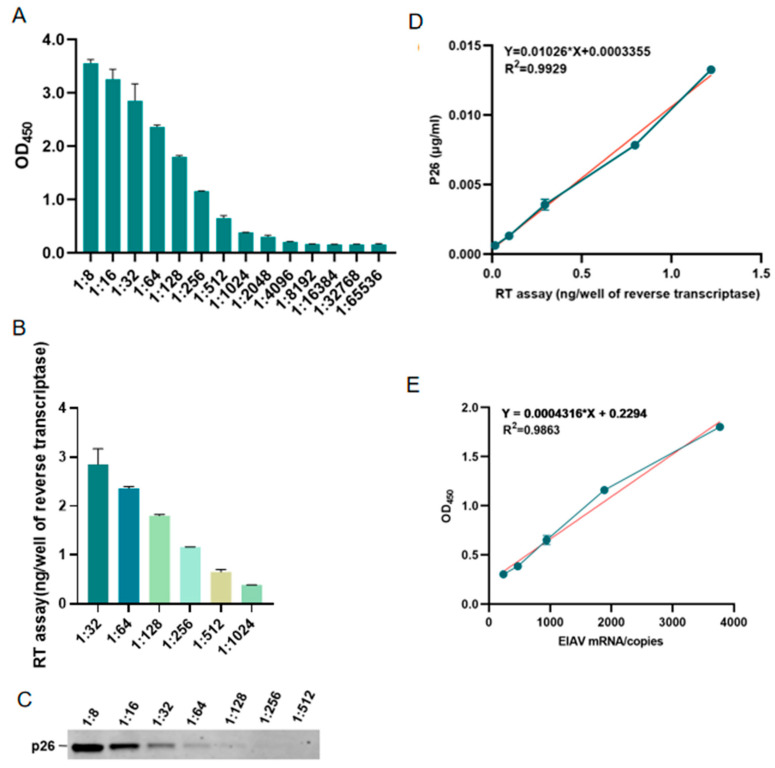
Comparison of the novel AC-ELISA and other methods. (**A**) A serially diluted EIAV-luc was quantified by AC-ELISA. (**B**) The EIAV-luc was serially diluted and used for reverse transcriptase activity. (**C**) The EIAV-luc was serially diluted and used for western blot detection of the p26 protein. (**D**) Correlation analysis between AC-ELISA and real-time qPCR detection of EIAV-luc. (**E**) Correlation analysis between AC-ELISA and real-time qPCR detection of EIAV-luc. Blue data points: Raw experimental measurements used to generate the calibration curve. Red regression line: Quantification of the relationship between Y-axis and X-axis.

Different methods were carried out for the detection of EIAV-luc, at an initial pseudo-virus particle concentration of 4.8 × 10^5^ genomic copies per microliter (copies/μL). The detectable limit was a 1:4096 dilution for the AC-ELISA (Figure 7A), but was a 1:1024 dilution for the reverse transcriptase assay (Figure 7B) and a 1:128 dilution for WB (Figure 7C). Comparative analysis revealed that the sensitivity thresholds of both previous methods fell below the detection limit of the AC-ELISA. As shown in Figure 7D, a good correlation was found between the concentration of p26 determined using the AC-ELISA and the amount of reverse transcriptase measured using the reverse transcriptase assay. The correlation coefficient (R^2^) between the two tests was 0.9929 for the quantification of EIAV-luc. For real-time qPCR, the amplification efficiency was 98.65%, with a linear dynamic range of 10^1^–10^7^ copies/μL (R^2^ = 0.997) (Figure 7D); sensitivity analysis established an LOD of 3.16 copies/μL and LOQ of 31.57 copies/μL, as described by the standard curve equation Y(CT Value) = −1.457 × ln(x[copies]) + 31.129. As shown in Figure 7D, a good correlation was found between the concentration of p26 determined using the AC-ELISA and the copies of rev gene measured using real-time qPCR. The correlation coefficient (R2) between the two tests was 0.9863 for the quantification of EIAV-luc range dilutions from 235–3771 copies, indicating that the AC-ELISA developed here was sensitive enough to monitor EIAV (Figure 7E). These results suggested that the developed AC-ELISA can quantify EIAV simply, rapidly, and accurately.

## 4. Discussion

The global prevalence of EIA means that, with increasingly frequent trade circulation, China still faces a high risk of EIAV introduction. Because EIAV exhibits a rapid rate of genetic variation [36,37,38,39,40], the relatively conserved p26 protein has been selected as the detection target for EIAV. Nevertheless, certain differences in the p26 protein exist among different virus strains [11,41]. Thus, there is an urgent need to develop a more universal AC-ELISA detection method for the inspection of imported and exported equine products, providing a strong safeguard against the invasion of EIA. Meanwhile, in laboratory research, EIAV, which is one of the simplest lentiviruses in terms of genomic structure, offers valuable insights for the development of an acquired immune deficiency syndrome (AIDS) vaccine through studies into its mechanisms of infection and immunity [22,23,36]. A rapid, simple, and specific monitoring method for EIAV is also crucial for such experimental studies. Although the reverse transcriptase assay is commonly used to screen for the presence of retrovirus, it is not specific to EIAV because reverse transcriptase activity can be detected in other lentiviruses [35]. Despite the existence of several methods for detecting EIAV in laboratory research, there is currently only limited technology of antigen testing capable of detecting multiple different EIAV strains [28,41]. This study describes the development of an AC-ELISA for the quantitative detection of EIAV p26 protein. This method utilizes a combination of specific mouse monoclonal antibody (1G11) and rabbit polyclonal antibody to ensure high specificity and sensitivity for the p26 protein in various EIAV strains and shows no cross-reactivity with other equine viral pathogens. Meanwhile, in our study, for the evaluation of the detection method, we utilized both recombinant p26 protein and viral particles as standard. The number of viral particles can be calculated based on the levels of the p26 antigen, like the quantitative relationship between p24 antigen and HIV particles. Furthermore, the 1G11 monoclonal antibody (mAb) was generated in our laboratory, targeting a linear epitope spanning 21 amino acid residues and exhibiting comprehensive cross-reactivity with all characterized EIAV isolates [33,42]. Otherwise, the use of pseudo-viruses instead of wild-type viruses for experimental analysis is primarily based on the lack of replication characteristics of the pseudo-virus and the issue of biosafety. Additionally, the production of pseudo-viruses is more convenient, as it can be accomplished using commonly available laboratory tools such as HEK293T cells, whereas wild-type viruses require the isolation of equine peripheral blood macrophages for propagation.

While the AC-ELISA developed in this study demonstrates promising specificity and sensitivity for detecting the EIAV capsid protein, several limitations must be acknowledged to guide further optimization and application. Firstly, the current assay relies on polyclonal rabbit antisera raised against the p26 capsid protein as the detection antibody, which has therefore not undergone purification. Although consistent with prior studies [43,44], the detection antibody in AC-ELISA was also derived from rabbit serum, and the antisera exhibited robust reactivity in our preliminary tests; the use of unpurified antibodies introduces the potential risks of non-specific binding or cross-reactivity, particularly when analyzing complex biological samples. To address this, future work will focus on affinity purification of the anti-p26 polyclonal antibodies to eliminate non-target immunoglobulins, thereby enhancing assay specificity. Additionally, direct conjugation of horseradish peroxidase (HRP) to the purified polyclonal antibodies is planned. This modification would streamline the assay workflow by eliminating the need for a secondary HRP-labeled anti-rabbit antibody, reducing both procedural complexity and assay time. Secondly, the absence of clinical EIAV samples in this study represents a significant constraint. Due to strict and effective Chinese control policies against EIA, we cannot obtain EIA-positive clinical samples. We acknowledge this is a major limitation of the current study. To resolve this, collaborative efforts with international laboratories or veterinary institutions in EIAV-endemic regions will be prioritized in a future study. Access to field samples (e.g., serum, plasma, or tissues from naturally infected horses) will enable rigorous evaluation of the AC-ELISA’s performance across diverse infection stages and viral loads, ensuring its utility in both diagnostic and epidemiological contexts. Thirdly, the current assay validation was limited to a narrow panel of EIAV strains, primarily vaccine-attenuated variants and a single American isolate. This raises concerns about the assay’s ability to detect genetically divergent or geographically distinct EIAV strains, such as virulent field isolates from Asia, Europe, or Africa. Given the high mutation rate of lentiviruses and the potential for epitope variability in the capsid protein, comprehensive validation against a broader spectrum of viral genotypes is essential. Future studies will aim to acquire additional EIAV isolates to assess the assay’s universality and adaptability.

Overall, this method not only improves the detection ability for different strains but also enables simultaneous monitoring of viral mutations, providing valuable data support for epidemiological studies and vaccine development. Furthermore, the antigen-capture ELISA has the advantages of being easy to operate and providing rapid detection, making it a valuable tool for laboratory research and clinical applications. In the future, by further optimizing detection conditions and expanding the range of antibodies, we hope to achieve precise detection of more EIAV strains. This will provide a deeper understanding of the biological characteristics of EIAV and its transmission.

## 5. Conclusions

In this study, we successfully developed a highly sensitive and specific antigen-capture ELISA (AC-ELISA) for the detection and quantification of the EIAV p26 protein, a relatively conserved and abundant structural protein critical for viral replication. By employing a dual-antibody system comprising monoclonal antibody 1G11 (capture) and polyclonal rabbit antisera (detector), the assay demonstrated exceptional performance with a detection limit of 1.95 ng/mL for purified p26 protein and 1:4096 dilution for pseudo-typed EIAV particles. The AC-ELISA exhibited no cross-reactivity with other major equine pathogens (EHV-1, EHV-4, EAV, EIV) and reliably detected diverse EIAV strains, including the Chinese virulent strain DLV34 and the UK reference strain. Comparative validation against western blot (WB) and reverse transcriptase (RT) assays revealed superior sensitivity, with AC-ELISA detecting viral antigens at 16-fold and 4-fold lower concentrations, respectively. The strong correlation (R^2^ = 0.9863) between p26 quantification by AC-ELISA and RT activity further confirmed its accuracy for viral load monitoring. This AC-ELISA addresses a critical gap in EIAV diagnostics by enabling cross-strain detection, thereby facilitating studies on viral evolution, vaccine efficacy, and epidemiological surveillance. Future efforts will focus on field validation using clinical samples and integration with portable detection platforms to enhance its utility in resource-limited settings. Our findings establish AC-ELISA as a robust, versatile method for advancing EIAV research and control strategies.

## Data Availability

The original contributions presented in this study are included in the article. Further inquiries can be directed to the corresponding authors.
